# Multisite Agricultural Veterans Affairs Farming and Recovery Mental Health Services (VA FARMS) Pilot Program: Protocol for a Responsive Mixed Methods Evaluation Study

**DOI:** 10.2196/40496

**Published:** 2023-01-06

**Authors:** Karen Besterman-Dahan, Wendy A Hathaway, Margeaux Chavez, Sarah Bradley, Tatiana Orozco, Vanessa Panaite, Jason Lind, Jessica Berumen

**Affiliations:** 1 Research Service James A Haley Veterans’ Hospital and Clinics Department of Veterans Affairs Tampa, FL United States; 2 Research Service North Florida/South Georgia Veterans Health System Department of Veterans Affairs Gainesville, FL United States

**Keywords:** veteran, evaluation, farming, farm, agriculture, nature, agricultural, mental health, mental health services, support, vocation, gardening, training, nature-based therapy, pilot program, posttraumatic stress disorder, PTSD, rehabilitation, reintegration

## Abstract

**Background:**

Veterans Affairs Farming and Recovery Mental Health Services (VA FARMS) is an innovative pilot program to provide supportive resources for veterans with interests in agricultural vocations. Implemented at 10 pilot sites, VA FARMS will provide mental health services and resources for veterans while supporting training in gardening and agriculture. As each pilot site project has unique goals, outreach strategies, and implementation efforts based on the local environment and veteran population, evaluating the pilot program provides a unique challenge for evaluators. This paper describes the protocol to evaluate VA FARMS, which was specifically designed to enable site variation by providing both site-specific and cross-site understanding of site implementation processes and outcomes.

**Objective:**

The objectives of this paper are to (1) describe the protocol used for evaluating VA FARMS, as an innovative Department of Veterans Affairs (VA) agriculturally based, mental health, and employment pilot program serving veterans at 10 pilot sites across the Veterans Health Administration enterprise; and (2) provide guidance to other evaluators assessing innovative programs.

**Methods:**

This evaluation uses the context, inputs, process, product (CIPP) model, which evaluates a program’s content and implementation to identify strengths and areas for improvement. Data collection will use a concurrent mixed methods approach. Quantitative data collection will involve quarterly program surveys, as well as three individual veteran participant surveys administered upon the veteran’s entrance and exit of the pilot program and 3 months postexit. Quantitative data will include baseline descriptive statistics and follow-up statistics on veteran health care utilization, health care status, and agriculture employment status. Qualitative data collection will include participant observation at each pilot site, and interviews with participants, staff, and community stakeholders. Qualitative data will provide insights about pilot program implementation processes, veterans’ experiences, and short-term participation outcomes.

**Results:**

Evaluation efforts began in December 2018 and are ongoing. Between October 2018 and September 2020, 494 veterans had enrolled in VA FARMS and 1326 veterans were reached through program activities such as demonstrations, informational presentations, and town-hall discussions. A total of 1623 community members and 655 VA employees were similarly reached by VA FARMS programming during that time. Data were collected between October 2018 and September 2020 in the form of 336 veteran surveys, 30 veteran interviews, 27 staff interviews, and 11 community partner interviews. Data analysis is expected to be completed by October 2022.

**Conclusions:**

This evaluation protocol will provide guidance to other evaluators assessing innovative programs. In its application to the VA FARMS pilot, the evaluation aims to add to existing literature on nature-based therapies and the rehabilitation outcomes of agricultural training programs for veterans. Results will provide programmatic insights on the implementation of pilot programs, along with needed improvements and modifications for the future expansion of VA FARMS and other veteran-focused agricultural programs.

**International Registered Report Identifier (IRRID):**

DERR1-10.2196/40496

## Introduction

### Background

In a health care organization as large and complex as the Veterans Health Administration (VHA), successful innovations are a strategic priority [1]. Health care service innovation and continuous quality improvement help meet the diverse needs of the over 9 million veterans who receive care from VHA’s 1293 health care facilities. The VHA’s primary goal is to deliver greater care and treatment choices “by improving experiences and outcomes” for veterans [2]. Rigorous evaluation ensures innovations are both fiscally responsible and support the public’s interest. Evaluative analysis is the foundation for data-driven decisions about the ultimate value of a practice [3]. Evaluating innovation is challenging because processes are new, and typical evaluation approaches are summative and emphasize averages, metrics, and short-term program outcomes [4]. This paper describes the protocol for evaluating an innovative VA agriculturally based mental health and employment pilot program, which serves veterans at 10 pilot project sites across the VHA enterprise. 

### Pilot Program

**Figure 1:**
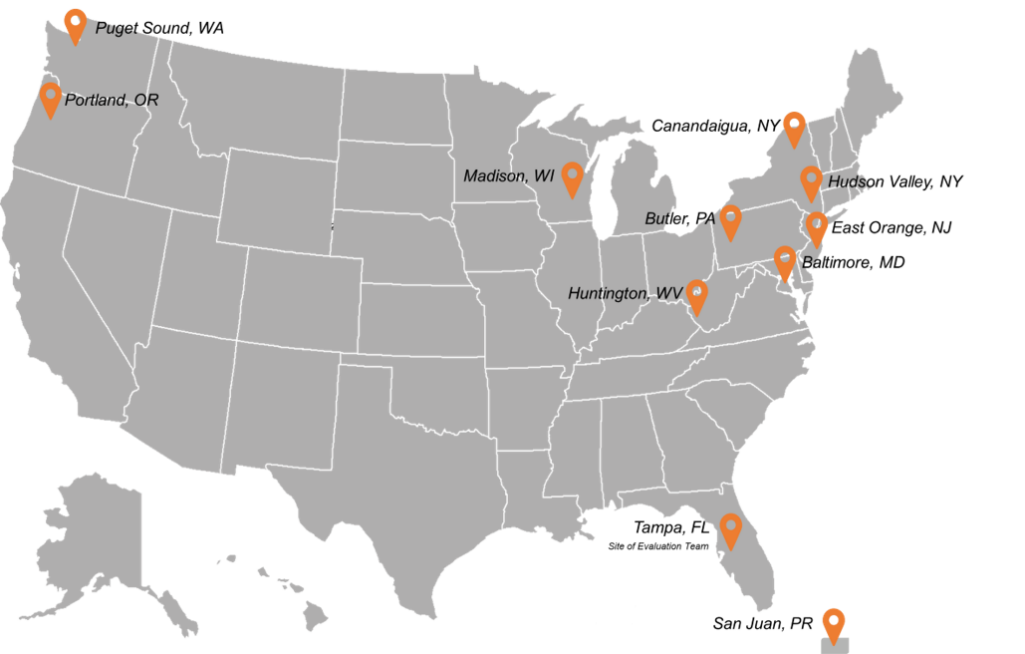
Map of funded pilot sites.

VHA’s Office of Rural Health (ORH) was established by US Congress in 2006 with a mission to improve health outcomes and increase access to care for the 2.7 million rural veterans enrolled in VHA care [4]. Given ORH’s mission, Senate Report (SR) 115-130 directed US $2,000,000 of funding to ORH to implement the Veterans Affairs Farming and Recovery Mental Health Services (VA FARMS) pilot program. In the spring of 2018, ORH partnered with VHA’s Office of Care Management and Social Work, Office of Mental Health and Suicide Prevention’s Therapeutic and Supported Employment section, Nutrition and Food Services, and Office of Community Engagement to develop a request for applications (RFA) that met the requirements outlined in SR 115-130. The RFA was open to VHA facilities and encouraged each applicant to partner with an existing community organization engaged in agricultural training. Guidelines for implementation were purposely broad so that each pilot site could tailor projects to meet the unique needs of their local population and the structure of their site. The RFA received 23 applications. Representatives from each partner office reviewed all applications with a standard set of criteria that examined feasibility, probability for success, and compliance with the congressional mandate. Ten applicants were chosen to participate in the pilot program (see Figure 1) [5]. Each VA FARMS pilot site project is unique in its design, goals and desired outcomes, duration, target veteran population, and programming. In general, pilot projects offer educational programming using a variety of modalities. These include virtual and in-person education, and some combination of classroom and hands-on project–based learning. Some pilot site projects include internships or other forms of direct job placement.

The goal of the VA FARMS pilot program is to provide supportive resources to veterans who desire agricultural-based treatment activities, specifically targeted to veterans diagnosed with posttraumatic stress disorder (PTSD) to complement evidence-based standard care approaches. Due to high rates of PTSD and suicide among veterans of all service eras [6], the VHA has prioritized the implementation of programs that connect veterans to critical mental health services and resources. The VHA also recognizes that there are many other social determinants of veteran health, such as economic stability and education. For this reason, programs that address multiple social determinants of health are of great value [7-9].

### Nature-Based Innovation for Veterans With PTSD and Mental Health Needs

The VA FARMS pilot program combines the benefits of nature-based therapies (NBTs) for veterans experiencing PTSD and other mental health issues with opportunities for vocational rehabilitation and training. This is in line with the Department of Veteran Affairs (VA)’s prioritization of the diagnosis and treatment of PTSD in veterans and their efforts to improve access to VA benefits to treat PTSD symptoms [10-12]. VA FARMS uses agritherapy, “an approach that incorporates mental health care and services with agricultural vocational training to support veterans’ behavioral and mental health needs” [13]. This is a novel approach that can positively influence community reintegration for veterans, especially those who face physical and psychological health issues, substance abuse, and unemployment [14].

Facilitation of community reintegration (CR) is part of the mission of the VA [2]. Veteran community reintegration refers to a military service member’s transition from active duty to participation in life outside of the military [15]. Key components of CR are (1) employment or other productive activities, (2) independent living, and (3) social relationships [15]. A substantial proportion of veterans report difficulties with CR following discharge from military service [16]. These difficulties are associated with poor social and family relationships, unemployment, financial strain, homelessness, and poor physical and mental health [17].

VHA’s commitment to veteran-centered care has improved veteran access to complementary and integrative health approaches [18]. One such example is NBT, which is an umbrella term for therapeutic approaches that incorporate nature as a key element of the therapeutic process [19]. These approaches can include outdoor programs, therapeutic landscapes, healing gardens, adventure-based counseling, and outdoor-based therapeutic recreation, as representative examples [11,20-23]. There are increasing numbers and types of outdoor programs that specifically offer different therapeutic opportunities for veterans to be immersed in natural environments [21]. Some veterans with PTSD symptoms seek to complement standard psychotherapy and medication treatments with NBTs [20,21,24-27]. For example, community gardens and other green spaces are recognized as safe, therapeutic community spaces [28,29]; they support reintegration by encouraging veterans to interact and socialize with other veterans and civilian community members while engaging in a personally and physically satisfying activity [11,30].

NBTs are not new treatment options for veterans. Historically, they were used to treat “shell shock,” war-related stress, traumatic stress, and PTSD among veterans returning from combat [14,31,32]. Although these NBTs are not considered standard care, there is some evidence that they are beneficial for the treatment of PTSD; however, much of this literature lacks measurement tools, precise descriptions of the therapeutic approach used, and clear distinctions between outdoor activities and NBT [23].

### Evaluation of the VA FARMS Pilot Program

Evaluating the VA FARMS pilot program will be challenging, in part because of its novelty. The rollout will be purposefully broad, allowing each pilot site’s goals, outreach, and implementation to differ based on the local environment and the unique needs of their veteran population. While mandated to provide training in agricultural vocations and access to behavioral health care services by licensed providers, each VA FARMS pilot site project is unique in its design and tailored to provide agricultural opportunities that meet local veteran needs. The evaluation protocol described herein will evaluate VA FARMS as a mechanism that supports tailored approaches to agricultural vocational training. The protocol allows for a site-specific and cross-site understanding of pilot project design and implementation processes. Programmatic data will provide a broad understanding of administrative logistics, promising practices, and modifications that may be needed to improve VA FARMS models. The protocol includes an assessment of the feasibility of measuring individual participant outcomes over time. By describing the protocol, this paper aims to provide guidance to other evaluators who are assessing innovative programs.

### Study Objectives

The specific objectives of this evaluation are to: (1) identify barriers and strategies to implement sustainable ORH-funded agritherapy pilot site projects; (2) identify best practices that can be used to inform future VA agritherapy programing; (3) describe barriers, benefits, and personal experiences of veterans who participated in VA FARMS programming; and (4) assess pilot program implementation processes, barriers, facilitators, strategies, and outcomes for providing mental health care referrals and developing pathways toward employment in the agricultural sector.

### Conceptual Framework

The context, inputs, process, product (CIPP) model [33] is well-suited to guide the evaluation of VA FARMS because it has been successfully used to evaluate training and educational programs [34] and innovations in dynamic real-world settings [35,36]. According to Stufflebeam and Shinkfield [35], the most important purpose of evaluation is not to “prove” but to “improve.” The CIPP model systematically evaluates a program’s content and implementation to identify strengths and areas for improvement. It enhances program effectiveness and supports program planning efforts [34]. Thus, this model is applicable to both formative and summative evaluations as it emphasizes “learning by doing” and promotes continuous program improvement [36].

**Figure 2:**
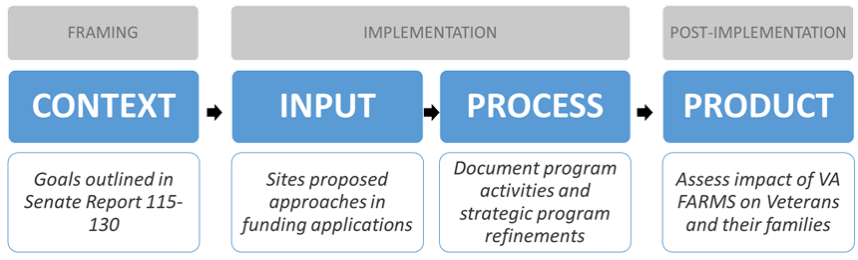
Scheme of application of the context, inputs, process, product (CIPP) framework to evaluation of the Veterans Affairs Farming and Recovery Mental Health Services (VA FARMS) pilot program.

The VA FARMS’s goals (*context*) were outlined in the congressional legislation. The pilot sites proposed unique approaches (*input*) in their funding applications to ORH. For example, they outlined budget and staffing needs, partnerships, and implementation strategies. The evaluation will record each pilot site program model and the degree to which this approach helped them achieve their goals; however, the primary focus will be to assess pilot program *processes* and *products* to suggest optimal models and promising practices. The process evaluation will document pilot program activities and strategic refinements. The product evaluation will assess the impact of participating in VA FARMS on veterans. Figure 2 schematically describes application of the CIPP framework in the VA FARMS evaluation.

## Methods

### Data Collection Procedures, Participants, and Recruitment

This 3-year evaluation will use a concurrent mixed methods approach to collect process and product data [37]. VA FARMS processes will be evaluated by collecting data about pilot program activities, implementation barriers, and modifications. These data will be collected through (1) quarterly surveys of project directors; (2) planned monthly *project status* conference calls between staff at individual pilot sites, ORH program management, and the evaluation team; (3) participant observation of activities at planned yearly site visits; and (4) *program implementation interviews* with VA FARMS pilot site staff and community stakeholders.

Products of VA FARMS will be evaluated by assessing the individual impact of participating in the pilot site projects. These data will be collected through (1) Individual *veteran participant surveys* administered by each pilot site at baseline (within 2 weeks of initial VA FARMS programming exposure), at exit (when a VA FARMS veteran participant ends their training activities), and at 3 months after the exit survey; and (2) *veteran voice interviews* with veteran participants who completed an *individual veteran participant survey* at baseline and/or exit. Interviews will be collected via telephone in years 2 and 3 of the pilot. The activities of data collection and the associated timeline are summarized in Table 1.

**Table 1:** Evaluation activities, methods, and timeline.

Method	Timeline	Participants	Procedure	Analysis	CIPP^a^ outcome
Project status conference calls	Years 1, 2, and 3	Program coordinators at all pilot sites	Collected monthly in a 1-hour meeting via teleconferencing technology	Content analysis of meeting minutes	Process evaluation
Individual veteran participant survey: baseline, exit, 3 months postexit	Years 1, 2, and 3	Veteran participants at all pilot sites	Collected on a rolling basis, distributed by pilot sites in paper-based or online format	Descriptive statistics: RMANOVA^b^, paired *t*-tests/ Wilcoxon signed-rank tests	Product evaluation
Quarterly program surveys	Years 1, 2, and 3	Program coordinators at all pilot sites	Collected every 3 months; administered online	Descriptive statistics	Process evaluation
Participant observation	Years 1, 2, and 3	Participants, staff, and stakeholders	Conducted yearly at annual site visits in person	Content analysis of field notes	Process evaluation
Implementation interviews	Years 1, 2, and 3	Year 1: Pilot site staff and community stakeholders; year 2: pilot site staff; year 3: pilot site staff and community stakeholders	Collected yearly in a 1-hour semistructured interview	Noticing, collecting, and thinking (NCT) analysis	Process evaluation
Veteran voice interviews	Years 1, 2, and 3	Veteran participants at all pilot sites	Conducted yearly in 30-minute semistructured interviews in person and via telephone	NCT analysis	Product evaluation

^a^CIPP: context, inputs, process, product.

^b^RMANOVA: repeated-measures analysis of variance.

### Data Collection Procedures

#### Individual Veteran Surveys

Individual veteran participant surveys (see Multimedia Appendix 1) will be offered in two formats: paper-based and web-based. Completed paper-based hard copies will either be submitted to the evaluation team via United States Postal Service or scanned and sent via encrypted email. Surveys submitted via the online platform Qualtrics [38] will be accessible only to evaluation staff and stored on a secure drive. Each individual veteran participant survey includes a 16-item demographic questionnaire and the following validated measures: (1) Military to Civilian Questionnaire (M2CQ) [39], a validated measure of veteran community reintegration; (2) PTSD Checklist–Military Version (PCL-M) [40], a validated PTSD self-report questionnaire that assesses 20 Diagnostic and Statistical Manual of Mental Disorders (DSM)-5 symptoms of PTSD; (3) Patient-Reported Outcomes Measurement Information System (PROMIS) Global Health (10) SF: Quality of Life [41], a validated self-report measure that assesses overall physical health, mental health, social health, pain, fatigue, and overall perceived quality of life; and (4) Work and Meaning Inventory (WAMI) [42], a validated self-report measure that assesses the personal meaning people draw from the work they perform, as well as how work broadens life purpose and contributes to the greater good.

Individual veteran participant surveys that are administered at exit and 3 months postexit will also include a 20-item satisfaction survey.

#### Quarterly Program Surveys

Throughout the pilot period, quarterly program data will be reported to ORH electronically. Awardee sites will report the number of veteran participants recruited, attrition, completion, number of referrals for mental health services, number of participants who gained employment in agriculture, type and amount of service use/referrals made, and number and type of outreach events (see Multimedia Appendix 2). The evaluation team will rely on site-reported numbers of referrals as we are not able to access participant medical records.

#### Participant Observation and Field Notes

Evaluators will attend annual site visits lasting approximately 2.5 days. Site visits will include participant observation of classes, demonstrations, and field activities. This method will allow evaluators to participate in VA FARMS activities and observe the day-to-day operations from both a programmatic and veteran participant perspective. A field notes template (see Multimedia Appendix 3) will capture evaluator impressions during site visits, and a debrief worksheet (see Multimedia Appendix 3) will allow evaluators to write a formal postvisit report.

#### Interviews With VA FARMS Staff, Community Stakeholders, and Participants

Interview guides will be developed iteratively based on pilot program goals and site-specific context, as well as evaluation survey results and conversations with VA FARMS program staff and ORH partners during monthly project status update calls. All interview guides will be based on 30-minute semistructured interviews. In Year 1, *implementation interviews* (See Multimedia Appendix 4) will be conducted in person during annual site visits with VA FARMS pilot program staff and community stakeholders. Interviews with pilot site staff will focus on start-up and implementation processes, including facilitators and barriers. Interviews with community stakeholders will document how community partnerships impacted program goals and veteran service provision. Also in year 1, evaluators will conduct in-person *veteran voice interviews* during annual site visits. Interviews will focus on veterans’ experiences and short-term participation outcomes (eg, service access, goal attainment, satisfaction).

In year 2, *implementation interviews* with VA FARMS pilot site staff will focus on VA FARMS program modifications and lessons learned that were driven by implementation needs and barriers. *Veteran voice interviews* (see Multimedia Appendix 5) will be conducted via telephone to capture the lived experiences, meaning, and intermediate outcomes (eg, new knowledge, increased skills, service access, goal attainment, satisfaction) of veterans who participated in a VA FARMS pilot program in year 1.

In year 3, final *implementation interviews* will be conducted with VA FARMS staff, administrative leadership at facilities hosting pilot sites, and community stakeholders to holistically describe barriers and facilitators to program implementation, changes to VA FARMS programming, outcomes of community partnerships, and long-term sustainability prospects. *Veteran voice interviews* will be conducted via telephone to capture the lived experiences, meaning, and long-term outcomes of participating in VA FARMS (eg, changes in attitudes or values, modified behavior, perceived improved condition).

### Participant Recruitment

Evaluation participants will be recruited from each of the 10 local VA FARMS pilot project sites. Pilot project site directors will be required to provide quarterly pilot program data as a condition of funding. All other evaluation activities will be voluntary. A convenience sample of pilot project staff, partners, and veteran participants of VA FARMS pilot projects will be invited to take part in *implementation interviews*. Participants will be recruited based on exit survey data where veterans will indicate a willingness to participate in an interview discussing their experiences in the VA FARMS pilot program at each site. In years 2 and 3, *veteran voice interview* participants will be recruited based on exit survey data where veterans will indicate a willingness to participate in an interview discussing their experiences in the VA FARMS pilot program at each site.

### Data Analysis

#### Quantitative Data Analysis

Descriptive statistics will be provided for all quantitative measures, which will include frequencies and percentages for categorical variables, as well as means, medians, standard deviations, and ranges for continuous variables. For variables measured over multiple time points, statistics will be provided both cumulatively and per time point (ie, quarterly or annually). Where appropriate, paired *t*-tests (ie, comparing change at two time points), Wilcoxon signed-rank tests (ie, nonparametric test to compare change at two time points when data are not normally distributed), or repeated-measures analysis of variance (RMANOVA) will be used to compare changes in continuous outcomes over multiple measurement time points.

To understand implementation processes (ie, pilot program activities and strategic refinements), quarterly pilot program surveys will be analyzed using descriptive statistics, and information will be presented both per quarter and cumulatively. In addition, data about veteran enrollments, outreach activities, and community partnerships will be collected to describe the number of participants who receive VA and non-VA health care and resource referrals, and the number of participants who became employed in agriculture.

To understand implementation products (ie, the impact of VA FARMS on veterans and their families), baseline descriptive statistics will be provided for veteran demographics, previous experience with gardening or agriculture, health care utilization, and PTSD and associated factors. Demographics will include gender, age, race/ethnicity, education, baseline employment and student status, military service branch, military service era, and service-connected disability rating. Previous experience with gardening or agriculture will be quantified in months. Health care utilization will include frequency of primary care visits, specialist visits, other provider visits, and hospital stays. Factors associated with PTSD will include reintegration, global physical health, and global mental health (encompassing pain, fatigue, depression, and overall quality of life).

In addition to baseline descriptive statistics, follow-up statistics on participant outcomes will be obtained from self-reports and validated measures (M2CQ, PROMIS, PCL-M). Veterans will report on health care utilization, PTSD, and PTSD-related symptoms. It is important to note that the selected measures are not diagnostic but allow veterans to self-report the presence of the 20 DSM-5 symptoms of PTSD. Descriptive statistics will be provided for scores at pilot program exit and at 3 months postexit. Additionally, RMANOVA, paired *t*-tests, or Wilcoxon signed-rank tests—depending on the data distribution and satisfaction of analysis assumptions such as normality and sphericity—will be performed to compare baseline to exit scores and to compare exit scores to 3 months postexit scores. Participants will also be asked about their agriculture employment status.

#### Qualitative Data Analysis

##### Monthly Project Status Updates and Field Notes

Note-taking templates will be created to structure notes from project status update calls and observations from site visits. Project status call templates will include sections to record information about reported implementation activities, successes, and challenges. Field note templates (see Multimedia Appendix 3) will include sections to record information about observed program activities, reflections, emerging questions and analyses, and future actions.

Data will be analyzed using a matrix analysis process [43]. Notes will be summarized and added to individual rows of a Microsoft Excel spreadsheet. Evaluators will read each summary and identify important concepts. These concepts will be summarized with a single word or short phrase called a “code.” Each Microsoft Excel column header will be labeled with a qualitative code. Summaries will be coded by typing a “1” in the cells that join rows (summaries) and columns (qualitative codes) to indicate that a piece of text is meaningfully connected to a specific concept. Evaluators will compare coding structures, discuss discrepancies, and reach consensus about the essential meaning of the data.

##### Interviews With VA FARMS Staff, Stakeholders, and Participants

Semistructured interviews with VA FARMS staff, community stakeholders, and veteran participants will be conducted primarily over the phone, except during site visits where interviews will be conducted in person. In both cases, two experienced qualitative evaluation team members will conduct the interviews in tandem, one as the primary facilitator and the other as the notetaker and timekeeper. Detailed notes and an overall summary of the interview will be completed using the interview summary template. This template will be used as a first-level analysis document that will be integrated into the principal analysis effort using ATLAS.ti v8.0 [44].

Data collection and analysis will occur concurrently allowing for insights from data analysis to iteratively guide subsequent data collection (eg, modification of interview questions). The detailed note summaries will be uploaded into ATLAS.ti v8.0. Interview data will be analyzed using the noticing, collecting, and thinking (NCT) analysis model [45]. As defined, the NCT consists of three basic components: noticing, collecting, and thinking about interesting things in the data. The NCT model will use coding structures, writing memos, process mapping, and diagramming to describe, categorize, and connect the data. This process will help to determine common themes, patterns, and inconsistencies related to the participants’ experiences, perceptions, and opinions. The qualitative team will systematically develop a code book (ie, operationalize codes and thematic categories) by meeting routinely to review ongoing coding results, resolve coding issues that arise, and discuss the development of thematic coding categories. Hyperlinks may be used to compare sections of text that occur in different interviews. Advanced search techniques including Boolean, code co-occurrence, and cross-tabulation searches will be used to sort and compare important pieces of text by VA FARMS staff, community stakeholders, and veteran participants.

### Ethical Considerations

This evaluation has been determined by the local VA Research and Development Service at the James A Haley Veterans’ Hospital to be a quality improvement project and will thus not be subject to the Institutional Review Board review for research [46]. Given this assessment, no written informed consent will be required. However, all participants, including veterans, VA FARMS staff, administrative leadership, and community stakeholders, will be verbally assured of confidentiality and will complete required VA verbal and written consent for use of photographs and audio recordings (VA Form 10-3203).

### Privacy and Security

Interviews will be audio-recorded with the permission of each participant, including veterans, VA FARMS staff, administrative leadership, and community stakeholders. All data will be kept in a secured folder behind the VHA firewall. The folder will only be accessible to members of the evaluation team. After interviews have been summarized, audio recordings will be deleted.

### Ensuring Inclusion and Accessibility

Interview guides and recruitment invitations will be drafted at a sixth-grade reading level using “plain language” principles for clear communication [47]. These materials will also be drafted in Spanish for participants at the Puerto Rico pilot site. Veterans with hearing impairment will be interviewed via email using tailored methods [48,49].

### Expert Advisory Board

An advisory board of subject matter experts (SMEs) will assist the evaluation team in quarterly meetings as well as on an as-needed basis. Advisory board participants will include SMEs in farm safety, PTSD, veteran farming programs, and disability and farming issues. They will assist with instrument development, face validity, pilot testing, and technical and agricultural issues that may arise during the evaluation, along with data collection and interpretation questions. Additionally, they will be available for general technical and programmatic issues that may arise for awardee sites. They will not see any data, and any information provided will be in aggregate or deidentified form.

## Results

In July 2018, a total of 10 sites were awarded with funding to implement their proposed pilot projects [5] and were subsequently enrolled in evaluation efforts starting in December 2018. Data analysis will be ongoing to fulfill quarterly and annual reporting. Overall findings are expected to be submitted for publication by October 2022. As of the end of September 2020, some data had been collected and analyzed (Tables 2 and 3).

**Table 2:** Implementation outcomes in 2019 and 2020.

Evaluation period	Number of veterans enrolled^a^	Number of veterans reached^b^	Number of communities reached^c^	Number of VA^d^ employees reached^e^
October 2018 to September 2019	229	1326	1623	655
October 2019 to September 2020	265	2341	1696	1816
Total	494	3667	3319	2435

^a^Total number of unique individual veterans enrolled in the VA FARMS pilot program in a fiscal year. These are veterans who had never been enrolled in the VA FARMS pilot program previously.

^b^Total number of veterans reached by the VA FARMS pilot program in a fiscal year. This number includes veterans who are not enrolled in VA FARMS but participated in pilot program activities such as demonstrations, informational presentations, and town-hall discussions.

^c^Total number of community members reached by the VA FARMS pilot program in a fiscal year. This includes face-to-face contact with people who are not enrolled in VA FARMS through demonstrations, informational presentations, town-hall discussions, etc.

^d^VA: Department of Veteran Affairs.

^e^Total number of VA employees reached by the VA FARMS pilot program in a fiscal year. This includes face-to-face contact with VA employees through demonstrations, presentations, grand rounds, etc.

**Table 3:** Data collection activities and outcomes October 2019 to September 2021.

Data collection activities	October 2018 to September 2019, n	October 2019 to September 2020, n	Total, N
Project status updates	114	32	146
Baseline surveys	191	59	250
Exit surveys	36	34	70
3-month follow-up surveys	7	11	18
Quarterly program surveys	36	36	72
Site visits	8	0	8
Pilot site staff interviews	17	10	27
Pilot site community partner interviews	11	0	11
Veteran voice interviews	15	15	30

## Discussion

### Projected Significance

Agricultural programs have been found to provide physical and behavioral health benefits to veterans who are struggling to transition from military to civilian life. European studies on the impact of horticulture and agriculture on the health and well-being of veterans and service members have found positive benefits for veteran participants with PTSD, including feeling relaxed, secure, and safe; having a sense of purpose; a decrease in PTSD symptoms; and learning new skills [50-52]. In the United States, findings on the benefits associated with farming and horticulture on veterans and service members are sparse, and primarily come from studies and evaluations of regional programs and populations that are veteran-focused. For example, an evaluation of a veteran-oriented community agricultural initiative in western Washington found that participation in the program contributed to improved mental, physical, and emotional health; increased vocational skills, community connectedness, interpersonal communication, a sense of satisfaction, and a sense of belonging; and helped decrease stigma surrounding veteran status [53-56]. A recent pilot horticultural therapy intervention showed significant improvements in reported stress, depressed mood, pain, and loneliness, and a decline in suicidal ideation in high-risk veterans [57]. Overall, findings from the United States are similar to those found in European studies, including promotion of the formation of trusted interpersonal relationships and community connectedness [30,31,56]; improved vocational skills [30,31,55,58]; improvement in pain, self-efficacy, and quality of life [58]; and better nutritional quality of diet [59].

Despite a long history of farming as therapy for veterans [11,14,52,60] and positive coverage in the news media [13,61], there is limited scientific evidence supporting the specific use of agritherapy for mental health and vocational rehabilitation in this population. For this reason, rigorous evaluation of the VA FARMS pilot program processes and products will be critical for understanding the benefits, barriers, and outcomes of implementing innovative programs within the VA system of care. This mixed methods evaluation protocol is designed to inform on diverse VA FARMS pilot projects with site-specific contexts for a rich understanding of program context and local definitions of successful reintegration for veterans. This will be critical given the many contextual factors known to influence CR in veterans [62-65]. Both the funding mechanism and evaluation design will allow for multiple models of VA FARMS to be implemented and is uniquely situated, as the VA is deeply aware of the multifaceted, heterogeneous needs of veterans [63,66,67]. Ideally, this will allow for multiple promising practices and programs to emerge, which can act as models for other VAs to choose from and tailor as they implement for their veteran communities. For example, pilot programs such as VA FARMS that follow an ecological approach provide opportunities for veterans to practice CR, increase self-efficacy, increase social support, coordinate with trusted community members or organizations, and meet in nonstigmatizing community locations have been found to facilitate veteran CR and program participation [62,68]. Many of the VA FARMS pilot site projects will include some or all of these elements and will be assessed through this multisite evaluation.

Notably, there are no validated quantitative measures that capture CR or rehabilitation outcomes of agricultural training programs in the civilian or veteran population [23]. The need for research focusing on CR outcome measures in rehabilitation-related studies and the study of CR outcomes was proposed by a VA Rehabilitation Research and Development Work Group on Community Reintegration [15,69]. This evaluation will also help to address identified gaps in the NBT literature, notably a lack of measurement tools and poor descriptions of the therapeutic approach used [23]. In this evaluation, PTSD and associated factors such as reintegration, global physical health, and global mental health (encompassing pain, fatigue, depression, and overall quality of life) will be measured at baseline, exit, and 3 months postexit using validated scales. These data, along with self-report of health care utilization (frequency of primary care visits, specialist visits, other provider visits, hospital stays) and agriculture employment status, will provide a basis for comparing participant outcomes from the individual pilot site projects and VA FARMS as a whole. In addition, individual pilot site project activities, including therapeutic approaches, will be clearly described and categorized through quarterly reporting as well as observation during site visits.

### Conclusions

This 3-year evaluation will employ a concurrent mixed methods approach and a CIPP model to collect process and product data. The evaluation will allow for a site-specific and cross-project understanding of project designs and implementation processes, as well as an assessment of the feasibility of measuring individual participant outcomes over time. Results of this evaluation will contribute rigorous evaluation findings of veteran agritherapy programs to the literature, an area that is notably sparse. Additionally, this evaluation will address the lack of measurement tools and poor descriptions of the therapeutic approach used in the NBT literature [23]. Programmatic insights will include a broad understanding of administration impacts, needed improvements, and modifications for expansion of VA FARMS models. Additionally, since this evaluation protocol will be designed to provide a rich understanding of contexts and local pilot site definitions of successful veteran reintegration, promising practices that emerge will be able to be applied to not only the implementation of VA FARMS models but also may be extended to other veteran-focused agricultural programs with consideration of CR and PTSD.

## Appendix

Multimedia Appendix 1 Participant surveys.

Multimedia Appendix 2 Quarterly program surveys.

Multimedia Appendix 3 Field notes template.

Multimedia Appendix 4 Implementation interview guides.

Multimedia Appendix 5 Veteran voice interview guides.

## Abbreviations

CIPP: context, inputs, process, product model
CR: community reintegration
DSM: Diagnostic and Statistical Manual of Mental Disorders
M2CQ: Military to Civilian Questionnaire
NBT: nature-based therapy
NCT: noticing, collecting, and thinking
ORH: Office of Rural Health
PCL-M: PTSD Checklist–Military Version
PROMIS: Patient-Reported Outcomes Measurement Information System
PTSD: posttraumatic stress disorder
RFA: request for applications
RMANOVA: repeated-measures analysis of variance
SME: subject matter expert
SR: Senate Report
VA: Veterans Affairs
VA FARMS: Veterans Affairs Farming and Recovery Mental Health Services
VHA: Veterans Health Administration
WAMI: Work and Meaning Inventory

## Data Availability

The data sets generated during and/or analyzed during the current study are not publicly available due to veteran confidentiality but are available from the corresponding author on reasonable request.
