# Supplementary material for: Multisite Agricultural Veterans Affairs Farming and Recovery Mental Health Services (VA FARMS) Pilot Program: Protocol for a Responsive Mixed Methods Evaluation Study
Source: JMIR Res Protoc. 2023 Jan 6;12:e40496. doi: 10.2196/40496 (PMC9862336; doi:10.2196/40496)
Supplement: Multimedia Appendix 1 [file resprot_v12i1e40496_app1.pdf]

VA FARMS [YEAR] Baseline Survey

Section 1: Information and Instructions

Thank you for taking this survey. We will use your answers to improve the program.

Why am I taking this survey?

We need feedback from participants like you to evaluate this program.

What can I expect?

This survey will help us understand how well you like this program, how it affects your life, and what changes could be made. You will answer questions about demographics, mental health, and quality of life. We will ask you to take some sections of this survey three times during your program. This survey should take about 30 minutes to complete.

What will you do with the insights you generate from this survey?

Insights will be used to determine future funding of similar programs.

I have security concerns.

All replies will be confidential.

Please return a copy of this survey to your program staff.

Please Contact Karen.Besterman-Dahan@va.gov with questions about the project and Wendy.Hathaway@va.gov with questions about the survey.

Section 2: Site

1. Indicate the site of the VA FARMS program you are working with.

Section 3: Demographics

Please note that your responses are CONFIDENTIAL. No one at your VA or program site will be able to connect your answers to your identity.

- Name (first and last):
- Email address:
- Phone number with area code:

Can we contact you for a brief 30-minute phone interview? If yes, please provide the best method to reach you to schedule the interview at your preferred time.

- No
- Yes. Preferred method to reach me to schedule the phone interview (phone or email): ____

Q1 How do you identify your gender?

- ○ Man
- ○ Woman
- ○ Other response, please specify ____

Q2 What is your age in Years? ____

Q3 Please indicate the ethnic category that best describes you (please check all that apply):

- ☐ Black, African American
- ☐ Asian (Chinese, Filipino, Japanese, Korean etc.)
- ☐ White, Caucasian
- ☐ Hispanic or Latino
- ☐ Native Hawaiian or other Pacific Islander
- ☐ American Indian or Alaskan Native
- ☐ Unknown
- ☐ Other, please specify ____

Q4 Please indicate your highest level of education:

- ○ Elementary school (1st-6th grade)
- ○ Middle school (7th-8th grade)
- ○ High school (9th-12th grade)
- ○ GED
- ○ Some College/Vocational School
- ○ Associate Degree (e.g. AA, AS)
- ○ Bachelor’s Degree (e.g. BA, AB, BS)
- ○ Graduate Degree (e.g. MA, MS, PhD)

Q5 Are you currently employed in the agricultural sector (not counting VA FARMS programming)?

- No
- Paid (temporary)
- Paid (permanent)
- Non-paid/volunteering

Q6 Indicate your employment status (please check all that apply):

- ☐ Employed part-time
- ☐ Employed full-time
- ☐ Self employed
- ☐ Homemaker
- ☐ Unemployed and looking
- ☐ Unemployed and NOT looking
- ☐ Retired
- ☐ Medically retired
- ☐ Volunteering
- ☐ Student (Part-time)
- ☐ Student (Full-time)

Q7 Please indicate your most current marital status:

- Married
- Separated
- Divorced
- Single/Never Married
- Widower/Widow
- Partner, not married
- Unknown

Q8 How much experience do you have in gardening or agriculture?

- 0-5 months
- 6 months – 2 years
- 25 months – 5 years
- >5 years

Q9 In which branch or branches of the military do/did you serve? (please check all that apply):

- ☐ Army
- ☐ Army National Guard
- ☐ Navy
- ☐ Air Force
- ☐ Air National Guard
- ☐ Marine Corps
- ☐ Coast Guard
- ☐ Other (e.g. the Public Health Service, Merchant Marines)

____

Q10 When did you serve on active duty in the U.S. Armed Forces? (please check all that apply):

- ☐ September 2001 or later
- ☐ August 1990 to August 2001 (includes Persian Gulf War)
- ☐ May 1975 to July 1990
- ☐ Vietnam era (August 1964 to April 1975)
- ☐ February 1955 to July 1964
- ☐ Korean War (July 1950 to January 1955)
- ☐ January 1947 to June 1950
- ☐ World War II (December 1941 to December 1946)
- ☐ November 1941 or earlier

Q11 Did you deploy in support of the U.S. Armed Forces?

- No
- Yes

Q12 If yes, were any of your deployments in a combat zone (persons serving in a war zone usually receive a combat tax zone exclusion, imminent danger pay, or hostile fire pay)?

- No
- Yes

Q13 Do you have a disability?

- No
- Yes- service connected- VA
- Yes- service connected- DOD
- Yes- not service connected

Q14 Do you have PTSD related symptoms?

This question was phrased to capture those participants without official PTSD diagnoses as well as to avoid documenting diagnostic health information.

- No
- Yes

Q15 Have you ever attended an appointment with a provider for PTSD related symptoms?

- No
- Yes
- N/A

Q16 Where was that appointment? (please check all that apply):

- ☐ DOD provider
- ☐ VA provider (including Vet Center)
- ☐ Community provider
- ☐ N/A

Section 4: M2CQ: Military to Civilian Questionnaire

This measure will help us understand your experiences after transitioning out of the military. Please read each question and then rate the amount of difficulty you have been having over the past 30 days. Please only *mark one choice per row.* **Over the past 30 days have you had difficulty with:**

	No difficulty	A little difficulty	Some difficulty	A lot of difficulty	Extreme Difficulty	NA
Over the past 30 days, have you had difficulty with:	0	1	2	3	4	99
1. Dealing with people you do not know well (such as acquaintance or stranger)?	0	1	2	3	4	
2. Making new friends?	0	1	2	3	4	
3. Keeping up friendships with people who have no military experience?	0	1	2	3	4	
4. Keeping up friendships with people who **have** military experience (including friends who are active duty or veterans)?	0	1	2	3	4	
5. Getting along with relatives (such as siblings, parents, grandparents, in laws and children not living at home)?	0	1	2	3	4	
6. Getting along with your spouse or partner (such as communicating, doing things together, enjoying his or her company)?	0	1	2	3	4	99
7. Getting along with your child or children (such as communicating, doing things together, enjoying his or her company)?	0	1	2	3	4	99
8. Finding or keeping a job (paid or non-paid or self-employment)?	0	1	2	3	4	99
9. Doing what you need to for work or school?	0	1	2	3	4	99
10. Taking care of your chores at home (such as housework, yard work, cooking, cleaning, shopping, errands)?	0	1	2	3	4	
11. Taking care of your health (such as exercising, sleeping, bathing, eating well, taking medications as needed)?	0	1	2	3	4	
12. Enjoying or making good use of free time?	0	1	2	3	4	
13. Taking part in community events or celebrations (for example festivals, PTA meetings, religious or other activities)?	0	1	2	3	4	
14. Feeling like you belong in "civilian" society?	0	1	2	3	4	
15. Confiding or sharing personal thoughts and feelings?	0	1	2	3	4	
16. Finding meaning or purpose in life?	0	1	2	3	4	

Section 5: PCL-M: PTSD Checklist Military Version

Below is a list of problems and complaints that Veterans sometimes have in response to stressful military experiences. Please read and *mark one choice per row* to indicate how much you have been bothered by that problem in the past month.

In the past month, how much you have been bothered by:

	Not at all	A little Bit	Moderately	Quite a Bit	Extremely
In the last month how much have you been bothered by:	1	2	3	4	5
1. Repeated, disturbing memories, thoughts, or images of a stressful military experience?	1	2	3	4	5
2. Repeated, disturbing dreams of a stressful military experience?	1	2	3	4	5
3. Suddenly acting or feeling as if a stressful military experience were happening again (as if you were reliving it)?	1	2	3	4	5
4. Feeling very upset when something reminded you of a stressful military experience?	1	2	3	4	5
5. Having physical reactions (e.g., heart pounding, trouble breathing, sweating) when something reminded you of a stressful military experience?	1	2	3	4	5
6. Avoiding thinking about or talking about a stressful military experience or avoiding having feelings related to it?	1	2	3	4	5
7. Avoiding activities or situations because they reminded you of a stressful military experience?	1	2	3	4	5
8. Trouble remembering important parts of a stressful military experience?	1	2	3	4	5
9. Loss of interest in activities that you used to enjoy?	1	2	3	4	5
10. Feeling distant or cut off from other people?	1	2	3	4	5
11. Feeling emotionally numb or being unable to have loving feelings for those close to you?	1	2	3	4	5
12. Feeling as if your future somehow will be cut short?	1	2	3	4	5
13. Trouble falling or staying asleep?	1	2	3	4	5
14. Feeling irritable or having angry outbursts?	1	2	3	4	5
15. Having difficulty concentrating?	1	2	3	4	5
16. Being “super alert” or watchful or on guard?	1	2	3	4	5
17. Feeling jumpy or easily startled?	1	2	3	4	5

Section 6: PROMIS Global Health (10) SF: Quality of Life

This measure will help us understand your general health. Please read each question and then respond to each item by *marking one choice per row*.

	Excellent	Very Good	Good	Fair	Poor
1. In general, would you say your health is:	5	4	3	2	1
2. In general, would you say your quality of life is:	5	4	3	2	1
3. In general, how would you rate your physical health?	5	4	3	2	1
4. In general, how would you rate your mental health, including your mood and your ability to think?	5	4	3	2	1
5. In general, how would you rate your satisfaction with your social activities and relationships?	5	4	3	2	1
6. In general, please rate how well you carry out your usual social activities and roles. (This includes activities at home, at work and in your community, and responsibilities as a parent, child, spouse, employee, friend, etc.)	5	4	3	2	1
	Completely	Mostly	Moderately	A little	Not at All
7. To what extent are you able to carry out your everyday physical activities such as walking, climbing stairs, carrying groceries, or moving a chair?	5	4	3	2	1
	Never	Rarely	Sometimes	Often	Always
8. How often have you been bothered by emotional problems such as feeling anxious, depressed or irritable?	5	4	3	2	1
	None	Mild	Moderate	Severe	Very Severe
9. How would you rate your fatigue on average?	5	4	3	2	1

10. How would you rate your pain on average? Select one answer.

0	1	2	3	4	5	6	7	8	9	10
☐	☐	☐	☐	☐	☐	☐	☐	☐	☐	☐

No Pain　　　　　　　　　　　　　　　　　　　　　　　Worst Pain Imaginable

Thank you for completing this survey!

Please feel free to contact us. with any questions or concerns. Karen Besterman-Dahan, PhD is leading this evaluation. She can be reached at Karen.Besterman-Dahan@va.gov

VA FARMS [YEAR] Exit and 3-Month Post Survey

Section 1: Information and Instructions

Thank you for taking this survey. We will use your answers to improve the program.

Why am I taking this survey?

We need feedback from participants like you to evaluate this program.

What can I expect?

This survey will help us understand how well you like this program, how it affects your life, and what changes could be made. You will answer questions about demographics, mental health, and quality of life. We will ask you to take some sections of this survey three times during your program. This survey should take about 30 minutes to complete.

What will you do with the information from this survey?

Insights will used to determine future funding of similar programs.

I have security concerns.

All replies will be confidential.

Please return copy of this survey to your program staff.

Please Contact Karen.Besterman-Dahan@va.gov with questions about the project and Wendy.Hathaway@va.gov with questions about the survey.

Section 2: Site

1. Indicate the site of the VA FARMS program you are working with.

Section 3: Demographics

Please note that your responses are CONFIDENTIAL. No one at your VA or program site will be able to connect your answers to your identity.

- Name (first and last):
- Email address:
- Phone number with area code:

Can we contact you for a brief 30-minute phone interview? If yes, please provide the best method to reach you to schedule the interview at your preferred time.

- No
- Yes. Preferred method to reach me to schedule the phone interview (phone or email): ____

Q1 Are you currently employed in the agricultural sector (not counting VA FARMS programming)?

- No
- Paid (temporary)
- Paid (permanent)
- Non-paid/volunteering

Section 4: M2CQ: Military to Civilian Questionnaire

This measure will help us understand your experiences after transitioning out of the military. Please read each question and then rate the amount of difficulty you have been having over the past 30 days. Please only *mark one choice per row.* **Over the past 30 days have you had difficulty with:**

	No difficulty	A little difficulty	Some difficulty	A lot of difficulty	Extreme Difficulty	NA
Over the past 30 days, have you had difficulty with:	0	1	2	3	4	99
17. Dealing with people you do not know well (such as acquaintance or stranger)?	0	1	2	3	4	
18. Making new friends?	0	1	2	3	4	
19. Keeping up friendships with people who have no military experience?	0	1	2	3	4	
20. Keeping up friendships with people who **have** military experience (including friends who are active duty or veterans)?	0	1	2	3	4	
21. Getting along with relatives (such as siblings, parents, grandparents, in laws and children not living at home)?	0	1	2	3	4	
22. Getting along with your spouse or partner (such as communicating, doing things together, enjoying his or her company)?	0	1	2	3	4	99
23. Getting along with your child or children (such as communicating, doing things together, enjoying his or her company)?	0	1	2	3	4	99
24. Finding or keeping a job (paid or non-paid or self-employment)?	0	1	2	3	4	99
25. Doing what you need to for work or school?	0	1	2	3	4	99
26. Taking care of your chores at home (such as housework, yard work, cooking, cleaning, shopping, errands)?	0	1	2	3	4	
27. Taking care of your health (such as exercising, sleeping, bathing, eating well, taking medications as needed)?	0	1	2	3	4	
28. Enjoying or making good use of free time?	0	1	2	3	4	
29. Taking part in community events or celebrations (for example festivals, PTA meetings, religious or other activities)?	0	1	2	3	4	
30. Feeling like you belong in "civilian" society?	0	1	2	3	4	
31. Confiding or sharing personal thoughts and feelings?	0	1	2	3	4	
32. Finding meaning or purpose in life?	0	1	2	3	4	

Section 5: PCL-M: PTSD Checklist Military Version

Below is a list of problems and complaints that Veterans sometimes have in response to stressful military experiences. Please read and *mark one choice per row* to indicate how much you have been bothered by that problem in the past month.

In the past month, how much you have been bothered by:

	Not at all	A little Bit	Moderately	Quite a Bit	Extremely
In the last month how much have you been bothered by:	1	2	3	4	5
1. Repeated, disturbing memories, thoughts, or images of a stressful military experience?	1	2	3	4	5
2. Repeated, disturbing dreams of a stressful military experience?	1	2	3	4	5
3. Suddenly acting or feeling as if a stressful military experience were happening again (as if you were reliving it)?	1	2	3	4	5
4. Feeling very upset when something reminded you of a stressful military experience?	1	2	3	4	5
5. Having physical reactions (e.g., heart pounding, trouble breathing, sweating) when something reminded you of a stressful military experience?	1	2	3	4	5
6. Avoiding thinking about or talking about a stressful military experience or avoiding having feelings related to it?	1	2	3	4	5
7. Avoiding activities or situations because they reminded you of a stressful military experience?	1	2	3	4	5
8. Trouble remembering important parts of a stressful military experience?	1	2	3	4	5
9. Loss of interest in activities that you used to enjoy?	1	2	3	4	5
10. Feeling distant or cut off from other people?	1	2	3	4	5
11. Feeling emotionally numb or being unable to have loving feelings for those close to you?	1	2	3	4	5
12. Feeling as if your future somehow will be cut short?	1	2	3	4	5
13. Trouble falling or staying asleep?	1	2	3	4	5
14. Feeling irritable or having angry outbursts?	1	2	3	4	5
15. Having difficulty concentrating?	1	2	3	4	5
16. Being “super alert” or watchful or on guard?	1	2	3	4	5
17. Feeling jumpy or easily startled?	1	2	3	4	5

Section 6: PROMIS Global Health (10) SF: Quality of Life

This measure will help us understand your general health. Please read each question and then respond to each item by *marking one choice per row*.

	Excellent	Very Good	Good	Fair	Poor
1. In general, would you say your health is:	5	4	3	2	1
2. In general, would you say your quality of life is:	5	4	3	2	1
3. In general, how would you rate your physical health?	5	4	3	2	1
4. In general, how would you rate your mental health, including your mood and your ability to think?	5	4	3	2	1
5. In general, how would you rate your satisfaction with your social activities and relationships?	5	4	3	2	1
6. In general, please rate how well you carry out your usual social activities and roles. (This includes activities at home, at work and in your community, and responsibilities as a parent, child, spouse, employee, friend, etc.)	5	4	3	2	1
	Completely	Mostly	Moderately	A little	Not at All
7. To what extent are you able to carry out your everyday physical activities such as walking, climbing stairs, carrying groceries, or moving a chair?	5	4	3	2	1
	Never	Rarely	Sometimes	Often	Always
8. How often have you been bothered by emotional problems such as feeling anxious, depressed or irritable?	5	4	3	2	1
	None	Mild	Moderate	Severe	Very Severe
9. How would you rate your fatigue on average?	5	4	3	2	1

10. How would you rate your pain on average? Select one answer.

0	1	2	3	4	5	6	7	8	9	10
☐	☐	☐	☐	☐	☐	☐	☐	☐	☐	☐

No Pain　　　　　　　　　　　　　　　　　　　　　　Worst Pain Imaginable

Section 7: The Work and Meaning Inventory

Please reflect on your experience in VA FARMS for this next group of questions. Work can mean a lot of different things to different people. The following items ask about how you see the role of work in your own life. Please honestly indicate how true each statement is for you and your work. Please read each question and then respond to each item by *marking one choice per row.*

	Absolutely Untrue	Mostly Untrue	Neither True nor Untrue	Mostly True	Absolutely True
1. I have found a meaningful career.	1	2	3	4	5
2. I view my work as contributing to my personal growth.	1	2	3	4	5
3. My work really makes no difference to the world.	1	2	3	4	5
4. I understand how my work contributes to my life's meaning.	1	2	3	4	5
5. I have a good sense of what makes my job meaningful.	1	2	3	4	5
6. I know my work makes a positive difference in the world.	1	2	3	4	5
7. My work helps me better understand myself.	1	2	3	4	5
8. I have discovered work that has a satisfying purpose.	1	2	3	4	5
9. My work helps me make sense of the world around me.	1	2	3	4	5
10. The work I do serves a greater purpose.	1	2	3	4	5

Section 8: Participant End of Program Satisfaction Survey

The purpose of this survey is to measure your satisfaction after participating in VA FARMS.

	Strongly Disagree	Disagree	Neither Agree or Disagree	Agree	Strongly Agree	N/A
1. I had a positive experience with the agricultural program.	1	2	3	4	5	99
2. I would recommend this program to other Veterans.	1	2	3	4	5	99
3. It was easy to get referrals to mental health services through the VA FARMS program.	1	2	3	4	5	99
4. It was easy to obtain referrals to other health services through the VA FARMS program.	1	2	3	4	5	99
5. I am satisfied with the treatment I received after being referred.	1	2	3	4	5	99
6. I am satisfied that the skills I learned through this program will help me gain employment in the agricultural/gardening fields.	1	2	3	4	5	99
7. This program helped me grow professionally.	1	2	3	4	5	99
8. The length of this program was appropriate to meet the intended learning goals.	1	2	3	4	5	99
9. I received the appropriate amount of assistance to be successful in this program.	1	2	3	4	5	99
10. I am satisfied with the teacher/trainers.	1	2	3	4	5	99
11. The teachers/trainers respected my needs.	1	2	3	4	5	99
12. I am satisfied with the amount of time staff teachers/trainers spent with me.	1	2	3	4	5	99
13. Knowledge/expertise of teachers/trainers aligned with program goals.	1	2	3	4	5	99
14. The location of this program was appropriate.	1	2	3	4	5	99
15. The necessary tools were available to me to achieve program goals.	1	2	3	4	5	99
16. This program provided opportunities to connect me with potential employers.	1	2	3	4	5	99
17. I felt that this program minimized health and safety risks.	1	2	3	4	5	99
18. This program allowed me to make connections with peers.	1	2	3	4	5	99
19. This program allowed me to connect with local community resources.	1	2	3	4	5	99

20. Is there anything else you would like to add about your experience?

Thank you for completing this survey!

Please feel free to contact us. with any questions or concerns. Karen Besterman-Dahan, PhD is leading this evaluation. She can be reached at Karen.Besterman-Dahan@va.gov
