# Supplementary material for: Multisite Agricultural Veterans Affairs Farming and Recovery Mental Health Services (VA FARMS) Pilot Program: Protocol for a Responsive Mixed Methods Evaluation Study
Source: JMIR Res Protoc. 2023 Jan 6;12:e40496. doi: 10.2196/40496 (PMC9862336; doi:10.2196/40496)
Supplement: Multimedia Appendix 2 [file resprot_v12i1e40496_app2.pdf]

Programmatic Data Collection Survey YEAR

Fiscal Year ___
Quarter ___
VA Site _________________

1) How many FTEs have been hired with this funding this quarter?

Participation Rate Data:

2) Total # of veterans SERVED in the VA FARMS program this quarter (participants who were previously enrolled in VA FARMS but who are still participating in program activities AND those who are newly enrolled this quarter).

3) Total # of UNIQUE veterans NEWLY ENROLLED in the VA FARMS program this quarter (participants who have never been enrolled in VA FARMS previously and who would receive a baseline survey).

4) What was VA FARMS reach this quarter (this includes face-to-face contact with people who are not enrolled in VA FARMS through program activities such as demonstrations, informational presentations, town-hall discussions etc.)?

    a. Total # veterans reached by VA FARMS this quarter.
    b. Total # community members reached by VA FARMS this quarter.
    c. Total # VA employees reached by VA FARMS this quarter.

Veteran Enrollee Demographic Data for this Quarter:

5) Total # veteran VA FARMS program participants who gained employment in agriculture not inclusive of VA FARMS programming activities (exclude experiences like: CWT [Compensated Work Therapy])?, TSES [Therapeutic & Supported Employment Services], Master gardening volunteer hours, internships or work experience built into the VA FARMS curriculum, etc.):
    a. Paid temporary
    b. Paid permanent
    c. Non-paid (volunteering, unpaid internships, etc.)

Outreach Activities

6) Describe VA FARMS program outreach activities this quarter (check all that apply):
    - Direct referral or waitlist follow-up contact
    - Paper-based marketing (flyers, posters, etc.)
    - Presentations in the community (community health fairs, VSOs, farmers markets)
    - Presentations in the VA (VA health fairs, service meetings, demonstrations, etc.)
    - Social media advertisement (Twitter, FaceBook, VA PULSE, Instagram, etc.)
    - Media outlets (radio, newspapers, magazines, newsletters, etc.)
    - Other

Programmatic Data Collection Survey YEAR

7) Describe the educational activities that have been offered to your VA FARMS program participants this quarter (check all that apply):
   - Advanced Agricultural Vocational Training (multi-week curriculum which focuses on vocational training in the field of agriculture; curriculum can include classroom education, project-based learning, hands on activities)
   - Agritherapy-Horticultural Therapy (multi-week programming with a curriculum focused on wellness through agricultural activities and meaningful work; curriculum can include classroom education, hands on activities)
   - Expert Guest Speakers
   - Field Trips
   - Internships (a period of work experience offered by an agricultural organization for a limited period of time)
   - Master Gardener (established multi-week program that trains volunteers in the science and art of gardening, so they can advise and educate the public about gardening and horticulture; programs are usually affiliated with state universities and extension offices and include classroom education, hands on training, and internships)
   - Online education (provision of educational resources such as webinars, virtual training modules, virtual classroom, etc.)
   - Peer-to-Peer education
   - Workshops (single to multi-day curriculum which focuses on a single topic of interest; curriculum can include classroom education and hands on activities)

Community Partnerships

8) Total # new FORMAL community partnerships established this quarter to support your VA FARMS program (MOAs [Memorandum of Agreement], MOUs [Memorandum of Understanding], Contracts).

9) With whom did you establish these new FORMAL community partnerships?

10) Total # new INFORMAL community partnerships established this quarter to support your VA FARMS program (this is any kind of networking partnerships with whom you do not have any formal MOAs, MOUs, or contracts).

11) Types of new INFORMAL community partnerships ESTABLISHED this quarter to support your VA FARMS program (select all that apply):
    - Businesses
    - Non-profit
    - Academic
    - Religious
    - Local VA Services or Programs
    - National VA Services or Programs
    - Local government (city, county, state)
    - Other federal government
    - Other agricultural organizations

Programmatic Data Collection Survey YEAR

- Other healthcare originations or services
- Other, please specify: _______________

12) Were any formal partnerships (MOAs, MOUs, Contracts) DISCONTINUED this quarter?
   a) NO
   b) YES; if yes, why was this formal partnership discontinued this quarter?

Mental Health Referrals

13) Total # veteran VA FARMS program participants who received Mental Health referrals this quarter (because of their participation in the VA FARMS).

14) Total # of Mental Health referrals given to veteran VA FARMS program participants this quarter (because of their participation in the VA FARMS).

15) Types of Mental Health referrals given this quarter (select all that apply):
   - Substance abuse
   - Counseling
   - Psychiatric services
   - Other, please specify: _____________

Other Referrals

16) Total # of other VA healthcare services referrals this quarter (because of their participation in the VA FARMS).

17) Total # of other community-based healthcare services referrals this quarter (because of their participation in the VA FARMS).

18) Total # of other non-healthcare referrals this quarter (because of their participation in the VA FARMS) (select all that apply):
   a) childcare
   b) benefits (e.g., education, social security, cemetery, etc.)
   c) food assistance (e.g., SNAP, WIC, foodbanks or pantries)
   d) housing (e.g., HUD/VASH, residential transitional housing)
   e) legal aid (e.g., Veteran Justice Court)
   f) transportation
   g) vocational rehab
   h) other, please specify:_______________________________________

19) If you need any assistance from the evaluation team, please describe. (This may include assistance with survey administration, data collection or reporting, etc.)

   Note: any matters involving budget management including Budget Change Requests (BCRs) should be referred to Office of Rural Health (ORH), Program Manager.

Multimedia Appendix: Quarterly Program Surveys
