# Supplementary material for: Multisite Agricultural Veterans Affairs Farming and Recovery Mental Health Services (VA FARMS) Pilot Program: Protocol for a Responsive Mixed Methods Evaluation Study
Source: JMIR Res Protoc. 2023 Jan 6;12:e40496. doi: 10.2196/40496 (PMC9862336; doi:10.2196/40496)
Supplement: Multimedia Appendix 3 [file resprot_v12i1e40496_app3.pdf]

Site Visit Information:

Site:	
Date:	
Observation #:	
Location:	
Observation Start Time:	
Observation End Time:	

Description of Activity

Who, what, where, when, why, and how | Stick to facts and direct description, avoid analysis in this section.

Reflections

Positionality, interpretations of observations, what went well, what could I do differently?

Emerging Questions & Analyses

Lines of inquiries, emerging themes, common narratives. Focus on our primary evaluation objectives: program infrastructure, program barriers and needs, program implementation strategies, program sustainability, population description, referrals processes, partnerships, etc.

Future Actions

Follow ups, next steps, other items of note, timeframes, and timelines
