# Supplementary material for: Multisite Agricultural Veterans Affairs Farming and Recovery Mental Health Services (VA FARMS) Pilot Program: Protocol for a Responsive Mixed Methods Evaluation Study
Source: JMIR Res Protoc. 2023 Jan 6;12:e40496. doi: 10.2196/40496 (PMC9862336; doi:10.2196/40496)
Supplement: Multimedia Appendix 4 [file resprot_v12i1e40496_app4.pdf]

Multimedia Appendix: Implementation Interviews

**Year 1:**

## Staff Interview Guide

**Introduction:** Thank you for meeting with us today to talk about your VA FARMS pilot program. This interview will take no more than one hour. Our goal today is to discuss the 1) program infrastructure, 2) your strategies for implementing the program, and 3) the population your program targets. Please know that this conversation is confidential. For the purposes of reporting, you will be identified as a "staff member". Before we begin, do we have your permission to audio-record this interview? Audio-recordings will be reviewed ONLY by the evaluation team. There is no right or wrong answer to any of the questions we ask you. Please answer openly and to the best of your ability about your experiences starting this VA FARMS program.

### Demographic Questions

1. What is your current position?
2. What is your role within VA FARMS program?
3. How long have you been involved in agritherapy or similar programs?

### Infrastructure

1. The program infrastructure was described as ____; would you add anything to this? (Example, contracting, hiring, staffing, resources, etc.)

2. In terms of your infrastructure, what do you need for the program to be successful?

3. [FOR PROGRAM DIRECTOR] What kind of partnerships did you develop to facilitate the VA FARMS program?
    a. How did these partnerships come about?

4. [FOR PROGRAM DIRECTOR] What processes or systems are in place to monitor program outcomes?

### Implementation

5. [FOR PROGRAM DIRECTOR] How has access to VA FARMS funding impacted the program?
    a. Who drove the development of this program?
    b. At what levels of leadership?

6. What has made program start-up more difficult? (example, funding, bureaucracy, contracts, etc.)

    a. How have you handled this? E.g., problem solving, networking
    b. What would make the process easier?
    c. What has made the process of implementation easy?

7. [FOR PROGRAM DIRECTOR] Describe the make-up of your team (such as different staff members, their roles, and the services they represent).

    a. Is the FTE [full time equivalents] for the staff adequate for the project? (e.g., Are people in collateral roles)? If not, how are you handling this?
    b. Is there anyone that you would have included in retrospect? (e.g., project coordinator, additional FTE)? Who?

8. What is your process for recruiting participants?

    a. How –if at all– has this changed since the program started?
    b. Can you describe your program's relationship with (Whole Health, CWT

9. What is the process for referring veterans into the VA FARMS program?
    a. How, if at all, is it noted in the veteran's record?

10. What is the process for referring participants to other VA services (Mental Health, CWT, or other) through this VA FARMS program?

    a. How, if at all, is it noted in the veteran's record?

**Population Characteristics**

11. In what ways do you think this program meets the needs of your clients?

    a. Does the VA FARMS Program help increase/support Veterans' access to Mental Health? How so? OR Why not?

12. In a year from now, how will you know your program has been successful?

## Community Partner Guide

**Introduction:** Thank you for meeting with us today to talk about your experience with the VA FARMS pilot program. This interview will take no more than one hour. Our goal today is to discuss your experiences as a community partner to [VA FARMS PILOT PROGRAM]. Please know that this conversation is confidential. For the purposes of reporting, you will be identified as a “community partner”. Before we begin, do we have your permission to audio-record this interview? Audio-recordings will be reviewed ONLY by the evaluation team. There is no right or wrong answer to any of the questions we ask you. Please answer openly and to the best of your ability about your experiences starting this VA FARMS program.

1. Tell me a little bit about [your organization]?

2. What is your role within [the organization]?

3. Describe how your partnership with [VA FARMS PILOT PROGRAM] came about?

    a. How often do you meet with [VA FARMS PILOT PROGRAM]?
    b. How do you communicate with [VA FARMS PILOT PROGRAM]?
    c. How involved are you in the decision-making process?

4. Have you experienced any challenges working with [VA FARMS PILOT PROGRAM]?

    d. What would make the process easier?
    e. What worked?

5. Is there anything else you need to fulfill your responsibilities for VA FARMS?

6. What resources and/or expertise does your partner organization provide to the [VA FARMS PILOT PROGRAM]?

7. How has partnering with your organization helped the [VA FARMS PILOT PROGRAM] fill any important gaps in their program or overcome barriers they have faced?

8. How does your [organization] meet the needs of veterans and/ or their family members?

9. What resources (employment, apprenticeships, mentorship, technical assistance, education, tours, etc.) does your organization provide to veterans who are participating in the [VA FARMS PILOT PROGRAM] specifically?

10. How will you know your work with this program has been successful?

## Year 2:

## Staff Interview Guide

**Interview Questions**

1. What changes did your site make to your original VA FARMS program during year 1 implementation?
    a. Why? Lessons learned? ORH? Leadership? Funding? Participant feedback? Change in community partnerships? Contracts/MOAs?

2. Tell us about how COVID-19 has impacted your VA FARMS program?
    a. Strategies for continuing VA FARMS activities during pandemic?
    b. Resources needed?

3. What does the next year of VA FARMS look like at your site?

4. Where are you in sustainability planning?

5. Who else should we speak to regarding your site's VA FARMS programming?

## Year 3:

## Staff Interview Guide

1. What is your biggest take-away from your work on VA FARMS?

2. Now that you have seen what it takes to implement VA FARMs at a local level, what are your thoughts about feasibly implementing the program more broadly?
    a. CBOCs [Community-Based Outpatient Clinics] or other VAMCs [Veterans Administration Medical Centers] in your VISN [Veterans Integrated Services Network]
    b. How do you see the VA incorporating this program enterprise-wide?
    c. Do you see it aligning with a specific service line or clinical program? Which ones, and why?

3. What resources are most needed to grow your program?
    a. Needs may be different in other locations. What essential resources do you think are critical to provide across the board, regardless of location? (e.g., FTE, position descriptions, P-card [purchasing card] for purchases, electricity and water, space, etc.)
    b. What about working with contracts? Any specific guidance or support from ORH or granting office to help navigate contracting processes?

4. What needs to happen to ensure future implementation and sustainability of VA FARMS?
   *(e.g. Technical assistance and support; Community of Practice; Awardee/Project conferences/Summit (for example, with other pilot sites, other resources like USDA; Community partner networking; etc.)*
   a. What could be done at your site?
   b. What could ORH do?
   c. What could the VA do at the organizational level?

5. What strategies have you used to successfully promote buy-in for this project with other clinical staff and hospital leadership?
   a. What hasn't worked so well and why?

6. Tell us about the impacts of COVID-19 on your VA FARMS program.
   a. Were and what were the positive outcomes of adapting to the pandemic restrictions?
   b. Were and what were the negative outcomes?
   c. How did your ability to adapt effect project sustainability?

7. What outcomes should be used to measure the success of the project?
   a. What would you document to show that Veterans were benefiting from the program?
   b. What about staff outcomes? How would people know you did your job?

8. How have you developed as a professional during your time on VA FARMS?

9. How has implementing this project changed your feelings about the VA Health Care System?

10. From your perspective, tell us about the impact of the VA FARMS program on the Veteran participants you served.

11. The VA FARMS program was designed to address mental health and vocational outcomes…
    a. Give us an example of how your VA FARMS program increased access to mental health services
    b. Give us an example of how it led to vocational opportunities in the agricultural sector.
    c. Were there any other important program outcomes not related to mental health or vocation? (e.g. changes in people's social skills, or quality of life, etc.)

12. Is there anything else you want to tell us about your experience so far with the VA FARMS program?
