# Supplementary material for: Multisite Agricultural Veterans Affairs Farming and Recovery Mental Health Services (VA FARMS) Pilot Program: Protocol for a Responsive Mixed Methods Evaluation Study
Source: JMIR Res Protoc. 2023 Jan 6;12:e40496. doi: 10.2196/40496 (PMC9862336; doi:10.2196/40496)
Supplement: Multimedia Appendix 5 [file resprot_v12i1e40496_app5.pdf]

Multimedia Appendix: Veteran Voice Interviews

Year 1:

Interview Procedures:

Interviews will be conducted by two VA Farms staff. Interviews will be conducted using the Veteran Voices interview guide. Interviews will be audio recorded upon consent of the Veteran participant. After each interview, detailed notes and an overall summary will be completed using the "Veteran Voices interview summary template." This summary template will be used to analyze the interviews using the matrix analysis approach outlined in the evaluation protocol.

VA FARMS

Veteran Voices Follow-up Interview Guide

Site:

Date of Interview:

Interviewers:

Participant Name:

1. **Tell me about your overall experience participating in the VA FARMS program** (Curriculum, benefits, time well spent, knowledge or skills, comradery with other Veterans).
    a. Did you complete the program? Tell me about that.
2. **What knowledge and skills did you gain through your participation?**
3. **How do you think your experience will be meaningful in the future?**
4. **What were your needs/goals/expectations coming into the VA FARMS program?**
5. **Do you feel the VA FARMS program met your needs/goals/expectations? Why or why not?**
    a. What could have been changed or done differently?
6. **In your experience, was there anything that made it difficult to participate in the VA FARMS program?** Such as transportation, family, class offerings, staff, location, interpersonal, time, work?
7. **Would you recommend the VA FARMS program to fellow Veterans?**

    a. Why/Why Not?

8. **Were you connected to additional programs or services through the VA FARMS program? (**within the VA or outside the VA, employment, experts, other community resources)?

    a. If so, what did that mean for you?

9. **Is there anything else you want to tell us about your VA FARMS experience?**

Year 2:

VA FARMS

Veteran Voices Follow-up Interview Guide

Site:

Date of Interview:

Interviewers:

Participant Name:

1. **Tell me about your overall experience participating in the VA FARMS program** (Curriculum, benefits, time well spent, knowledge or skills, comradery with other Veterans).

    a. How were you referred to the program/How did you find out about the program?
    b. Tell me about the VA FARMS curriculum you participated in.

2. **Did COVID-19 affect your participation in VA FARMS?**
    a. Did/how did you participate in VA FARMS during COVID-19?
    b. Did you participant in virtual curriculum? Access, benefits, appropriateness, and quality of virtual offerings?
    c. Did participating in VA FARMS have an effect on your experience through the pandemic?

3. **What were your needs/goals/expectations coming into the VA FARMS program?**

    a. Do you feel the VA FARMS program met your needs/goals/expectations? Why?
    b. Is there anything that could have been changed or done differently?

4. **What knowledge and skills did you gain through your participation?**

a. How might you use these skills in the future?

5. In your experience, was there anything that made it difficult to participate in the VA FARMS program?

a. Transportation, access to technology, family, class offerings, staff, location, interpersonal, time, work?
b. Anything that made it easy for you to participate in the VA FARMS program?

6. **Were you connected to additional programs or services through the VA FARMS program?**

a. Within the VA or outside the VA, employment, experts, other community resources?
b. Friendships or impact on family.
c. If so, what did that mean for you?

7. Would you recommend the VA FARMS program to fellow Veterans?

8. Is there anything else you want to tell us about your VA FARMS experience?

Year 3:

VA FARMS Veteran Voices Follow-up Interview Guide

Site:

Date of Interview:

Interviewers:

Participant Name:

1. **How did you find out about your program?**

2. Tell me a little bit about the program you attended. For example, what did you learn and do?

3. What did it mean for you to work with other Veterans in your VA FARMS project?

4. How did taking part in VA FARMS affect your health?

   a. How about your mental health?
   b. How about your stress levels?

5. **Has this program changed the way you think about your health and health care needs? How so?**

   a. How about prioritizing free time or leisure activities?
   b. How about the importance of reducing your stress levels?

6. **How has taking part in the program affected your social relationships or sense of community?**

   a. How about your family?
   b. How about at work?
   c. How about relationships with people in other places that are important to your (your church, volunteer organizations, neighborhood, etc.)

7. **How has this program affected the way your view your abilities or knowledge base?**

8. **How have you used the information or skills gained through VA FARMS?**

   a. What about plans for in the future?

9. **How did COVID-19 affect your participation in VA FARMS, if at all?**

   a. Did taking part in VA FARMS influence your experience through the pandemic?

10. **Has taking part in this program changed your feelings about the VA Health Care System? How so?**

    a. What should we do to increase the positive outcomes of this program for Veterans?

11. **Is there anything else you want to tell us about your VA FARMS experience?**
